# Alleviation of DSS-induced colitis by Meconopsis polysaccharides correlated with reduced PI3K/AKT signaling and gut microbiome diversity

**DOI:** 10.3389/fphar.2025.1459668

**Published:** 2025-02-12

**Authors:** Jun Dai, Weidong Wang, Fangfang He, Yujuan Wang, Denglang Zou

**Affiliations:** ^1^ Engineering technology research center of Plant Cell Engineering, West AnHui University, Lu’an, China; ^2^ College of Pharmacy, Hubei University of Chinese Medicine, Wuhan, China

**Keywords:** Meconopsis polysaccharides, DSS-induced colitis, PI3K/Akt signaling pathway, gut microbiome diversity, short-chain fatty acids

## Abstract

**Introduction:**

Inflammatory bowel disease (IBD) is a recurrent gastrointestinal disorder that significantly impacts patients’ quality of life globally. This study focuses on the polysaccharides (MP) extracted from *Meconopsis integrifolia*, to investigate its role in alleviating DSS (dextran sulfate sodium)-induced colitis in mice.

**Methods:**

The study commenced with a comprehensive chemical characterization of *Meconopsis polysaccharides*. Subsequently, the colitis-alleviating activity of MP was validated through *in vivo* experiments.

**Results:**

The results revealed that MP is primarily composed of ten monosaccharides, exhibits good thermal stability, and has a relatively uniform molecular weight distribution. *In vivo* experiments demonstrated that MP significantly mitigated DSS-induced weight loss, increased DAI, colon shortening, and tissue damage in mice. Furthermore, MP reduced the levels of inflammatory cytokines such as IL-1β, TNF-α, and IL-6 in serum. Mechanistically, MP exerted its anti-inflammatory effects by inhibiting the activation of the PI3K/AKT signaling pathway. Additionally, MP promoted gut microbiota diversity and regulated SCFA concentrations, contributing to an improved intestinal microenvironment and alleviation of colitis symptoms.

**Discussion:**

Our findings highlight the superior effectiveness of *Meconopsis polysaccharides* in alleviating DSS-induced colitis and open new avenues for targeted therapeutic strategies in the treatment of IBD.

## 1 Introduction

Inflammatory bowel disease (IBD), typically composed of ulcerative colitis and Crohn’s disease, is a recurrent gastrointestinal disease that can be physically and psychologically distressing (Baumgart and Carding, 2007; Strober et al., 2007; Roda et al., 2020). The incidence and progression of IBD vary across regions, with western countries reporting a higher prevalence. For instance, in the United States, the prevalence of IBD has been estimated at approximately 1.3% of the population (Kaplan and Windsor, 2021). In recent decades, the prevalence of IBD has also been on the rise in Eastern and Latin American countries, likely due to economic and demographic growth, further straining global healthcare systems (Colitis-Pathophysiology and Jurenka, 2003). IBD is a chronic idiopathic intestinal disease with an unclear cause, encompassing a complex interplay of gut microbiota, host genetic susceptibility, environmental factors, and aberrant immunology. In most cases, IBD patients exhibit variable degrees of colitis, accompanied by different types of epithelial barrier disruption (Du and Ha, 2020).

The primary treatments for IBD include immunomodulators and, in severe cases, surgical intervention (Kaplan and Windsor, 2021). Recently, new treatment options for IBD have emerged, such as JAK inhibitors, Anti-IL-12/23 agents, anti-TNF-α agents, and S1PR modulators, which are approved by FDA, EMA, *etc.*, and marketed for this indication (Adolph et al., 2022; Pittayanon et al., 2020). The primary treatments for IBD currently approved by the Food and Drug Administration (FDA) encompass a range of therapeutic modalities, each with distinct mechanisms of action. Immunomodulators, such as azathioprine and 6-mercaptopurine, work by suppressing the immune system to reduce inflammation (Ordas et al., 2012), Amino salicylates, like mesalamine, are anti-inflammatory agents that directly target the gut mucosa (Hanauer, 2006).

*Meconopsis integrifolia* is a distinctive species of plant, endemic to the Qinghai-Tibetan Plateau and neighboring regions, flourishing at elevations between 2,700 and 5,100 m in specific areas of China (Li et al., 2024). With a rich medicinal application history in traditional Tibetan medicine, it has been utilized to treat a plethora of ailments including hepatitis, pneumonia, inflammation, and pain (Li et al., 2020). The leaves alleviate stomach acid reflux when harvested before flowering, and the flowers reduce fever, mitigate inflammation, and assist in fracture healing, highlighting its anti-inflammatory potential (Zhou et al., 2013). The noted therapeutic attributes and historical medicinal applications of Meconopsis have led to increased scholarly attention, particularly towards its anti-inflammatory capacities (Guo et al., 2016). According to our preliminary, Polysaccharides, being complex carbohydrates found in numerous living organisms, possess a wide array of biological functions, including immunomodulatory and anti-inflammatory activities. This makes them promising candidates for the development of therapeutic agents targeting inflammatory conditions. In particular, polysaccharides derived from *Meconopsis integrifolia* may exhibit anti-inflammatory effects due to their potential to modulate immune responses and reduce inflammation, as suggested by preliminary studies.

Polysaccharides are important macromolecules found in nearly all living organisms and possess crucial biological functions (Hou et al., 2020; Yin et al., 2020; Niu et al., 2021). They are increasingly attracting attention due to their wide range of pharmacological and biological activities, including immunomodulatory, antitumor, antimicrobial, anticoagulant, antioxidant, antiviral, hypoglycemia, and antidiabetic properties, making them one of the most promising candidates in the field of biomedicine and pharmaceuticals (Yi et al., 2020; Leong et al., 2021; Fernandes and Coimbra, 2023). Polysaccharides can be gained from lots of origins, containing microorganisms, plants, animals, and algae. Because of their physicochemical features, they are susceptible to chemical and physical changes that improve their properties. This is the fundamental notion for their wide range of uses in biomedical and pharmaceutical industries.

This study comprehensively investigated the potential of Meconopsis polysaccharides (MP) to alleviate colitis induced by dextran sulfate sodium (DSS) in mice. Firstly, the Meconopsis polysaccharides was primarily characterized by chemical composition, homogeneity molecular weight, monosaccharide composition and thermal analysis. Then, its colitis alleviation activity was confirmed by *in vitro* experiments. The action mechanism was further primarily reveled by cell model and gut microbiota diversity. And it was found that Meconopsis polysaccharides could alleviate colitis induced by DSS via the regulation of PI3K/AKT signaling pathway. In addition, it alleviated the advancement of colitis by enhancing the regulation of gut diversity and concentration of short-chain fatty acids (SCFAs). Our results emphasize the outstangding efficacy of Meconopsis polysaccharides in relieving DSS-induced colitis and open up new way for potential therapy IBD.

## 2 Materials and methods

### 2.1 Chemical composition analysis

The content of uronic acid was determined using m-hydroxybenzene colorimetry method, with glucuronic acid employed as standard (Wang et al., 2015). The content of total sugar was quantified using phenol-sulfuric acid colorimetric assay (Laier Biotechnology Co., Ltd, Hefei, China), and D-glucose was employed as standard. The content of protein was measured using BCA assay (bicinchoninic acid assay), and bovine serum albumin was employed as standard (Merck, Germany).

### 2.2 UV-vis, FT-IR and CD spectroscopy analysis

The MP (1 mg/mL) aqueous solution was scanned with a UV-Vis spectrophotometer (JASCO V-550, JASCO., Kyoto, Japan) in the range 200 nm–700 nm. The sample mixture was prepared by MP (1 mg) and dried KBr (100 mg), the mixture was pressed into tablets. The MP FT-IR spectra was scanned via a FT-IR-4600 spectrophotometer (JASCO., Kyoto, Japan), and the scan range was set from 4,000 to 400 cm^-1^. The CD (circular dichroism) spectra was determined by J-180CD (JASCO, Kyoto, Japan) spectropolarimeter at 1.0 mg/mL MP solutions. The CD result was accumulated three scans with a rate of 50 nm/min. The slit width was set at 1 nm with 1 s for time constant. The CD spectra was obtained from 190 to 260 nm with a 1-nm interval.

### 2.3 Homogeneity molecular weight analysis

The MP molecular weight (Mw) and homogeneity were assessed using HPGPC (high performance gel permeation chromatography) on an Agilent 1,260 series HPLC(NYSEA, United States) with a RID detector (refractive index detector) and a G4000 SWXL column (7.8 × 300 mm, Tosoh Biosep, Japan). The specific experimental conditions were as outlined below: MP (3.0 mg) was mixed with 1 mL KH_2_PO_4_ (0.02 M). The temperature for column and RID was preserved at 35°C. The loading volume was 10 μL. It was eluted by 0.02 M KH_2_PO_4_ with a flow rate of 0.8 mL/min. The calibration curve was produced by plotting retention time against dextran standards. Subsequently, the MP molecular weight was measured from the produced calibration curve.

### 2.4 Monosaccharide composition analysis

The MP sample (5 mg) was hydrolyzed with 4 mL TFA (trifluoroacetic acid) at a concentration of 2 M under the temperature of 110°C for 4 h. The hydrolysate was then derivatized by PMP. At last, the derivatized MP sample was identified by ultra performance liquid chromatography (UPLC) analysis on a 1,260 Agilent with a CORTECS UPLC C18 column (2.1 × 100 mm, 1.6 μm) at 250 nm. The system temperature was set at 30°C and the loading volume was 3 μL. 50 mM Ammonium acetate (A) and acetonitrile (B) were used as mobile phase with a flow rate of 0.3 mL/min. The 10 monosaccharide standards, including Man, Rib, Rha, GlcA, GalA, Glc, Gal, Xyl, Ara and Fuc, were proceeded under the same conditions.

### 2.5 Thermal analysis

The thermal properties of the MP were analyzed by a TGA analyzer (DTG-60A, Shimadzu Co., Japan) through differential scanning calorimetry. The MP sample was heated at 35°C–600°C at 10°C/min for 40 mL/min in a platinum crucible under a flowing nitrogen atmosphere.

### 2.6 Animals

C57BL/6 mice, aged 6–9 weeks, were sourced from the Hubei Provincial Center for Disease Control and Prevention (Wuhan, China). The mice were placed in plastic cages under controlled conditions, with temperatures maintained between 24°C–26°C (license number SCXK 2020-0005). They had unrestricted access to water and food in a SPF (specific pathogen-free) with a standard light (12 h)/dark (12 h) cycle.

### 2.7 Induction and treatment of ulcerative colitis

To establish DSS-induced ulcerative colitis mouse models, C57BL/6 mice were administered 3.5% DSS (dextran sulfate sodium salt) daily for seven consecutive days. For assessing the protective effects of MP in colitis mice, the animals were randomly assigned to three groups: Control, model, and MP (200.0 mg/kg). The control and model groups received the same dose of solvent orally (0.2 mL). The MP group received preventative medication via oral gavage for 7 days, with MP dissolved in water before administration.

### 2.8 Disease activity index (DAI)

Daily monitoring of each mouse encompassed assessments of body weight, stool consistency, morbidity, and hematochezia. DAI scores were calculated using a standardized assay. Evaluation criteria included, but were not limited to, the degree of inflammatory cell infiltration, mucosal integrity, and glandular structural disorder. Each evaluation criterion was assigned a different score based on the degree of pathology, typically ranging from 0-3 or 0-4, with 0 indicating no pathology and the highest score indicating the most severe pathology.

### 2.9 Enzyme-linked immunosorbent assay

After a 12-hour fasting period and anesthesia, blood was obtained from the mice. The blood was left at room temperature for 2–3 h and subsequently centrifuged for 15 min with 3,000 rpm under 4°C. The supernatant, representing the mouse serum, was used to measure levels of MDA (Malondialdehyde), IL-1β (interleukin-1β), T-SOD (total Superoxide Dismutase), TNF-α (tumor necrosis factor-α), and IL-6 (interleukin-6). Freshly prepared serum samples were used for the assays. ELISA kits were sourced from Bioswamp (Wuhan, China).

### 2.10 Histological scores

The produced section of distal colon was fixed with 4% paraformaldehyde for 24 h, next underwent dehydration, paraffin embedding, sectioning, and staining with H&E (hematoxylin and eosin).

### 2.11 Western blotting

Colon tissues cells were lysed in RIPA buffer on ice with 1 mM PMSF for 10 min to obtain the protein lysate. The supernatant was centrifugated at 4°C with 12,000 rpm for 15 min, and the protein content was determined by BCA Protein Assay Kit (Prilley Gene Technology Co., Ltd, Beijing China). After the SDS-PAGE separation, the extracted protein samples were put onto PVDF membranes. The Protein Free Rapid Blocking Buffer was employed to block these PVDF membranes for 10 min. Then, the targeted primary antibodies (1:1,000) were incubated with the blocked membranes overnight at 4°C, and then the corresponding secondary antibodies (1:5,000) were further incubated for 1.5 h on the subsequent day. Finally, the bands were visualized using ChemiDoc XRS+ (sourced from Bio-Rad, CA, United States). Both primary and secondary antibodies used in the experiment were purchased from Cell Signaling Technology (Massachusetts, United States).

### 2.12 DNA extraction and 16S ribosome RNA V3∼V4 region sequencing

The total genomic DNA were extracted from the fecal samples (1.5 g) by DNA Kit. For V3-V4 region gene amplification from 16S rRNA, Q5^®^ High-Fidelity DNA Polymerase was employed. The forward primer sequence was 5′-ACT​CCT​ACG​GGA​GGC​AGC​A-3′ and reward primer sequence was 5′-ACT​CCT​ACG​GGA​GGC​AGC​A-3′. For multiplex sequencing, 7-bp barcodes with sample specificity were added to the primers. After purification, the resultant amplicons were measured using Quant-iT PicoGreen dsDNA Assay Kit (Nuowei Biotechnology Co., Ltd, Beijing China). A pair-end 2 × 250 bp sequencing was performed under Illlumina MiSeq platform. The corresponding 16S rRNA sequencing data has been deposited in Sequence Read Archive (SRA) of NCBI (The accession number is SRP519212: PRJNA1134356, https://submit.ncbi.nlm.nih.gov/subs/sra/).

Microbiome bioinformatics analysis was conducted via QIIME2 (Quantitative Insights Into Microbial Ecology). The obtained sequencing data was assembled and aligned according to the SILVA Release 132 Database. OTUs (operational taxonomic units) were aligned using MAFFT to build a phylogeny with fasttree2. α-Diversity index, including Simpson, Chao1, ACE, and Shannon indices, were used for comparison. The sample type used was fresh feces from mice, with a quantity of five mice per group. β-Diversity was analyzed using PCoA, PERMANOVA and ANOSIM. LEfSe were employed with the default parameters to identify specific taxa microbes among groups.

### 2.13 Content detection of SCFAs

After homogenization with 0.5 mL of water and 0.1 g of glass beads, the samples were centrifugated at 4°C with 12,000 rpm for 10 min. Subsequently, 0.2 mL of the supernatant was further extracted according to the standard procedure. The extracts were subjected to GC-MS analysis after centrifugation.

## 3 Results

### 3.1 Chemical composition

Evaluation of chemical composition showed that the content of uronic acid, the content of protein and the content of total sugar for MP was 9.39%, 14.62% and 60.79%, respectively. It was indicated that MP was a typical neutral polysaccharide.

### 3.2 UV-vis spectroscopy

**FIGURE 1:**
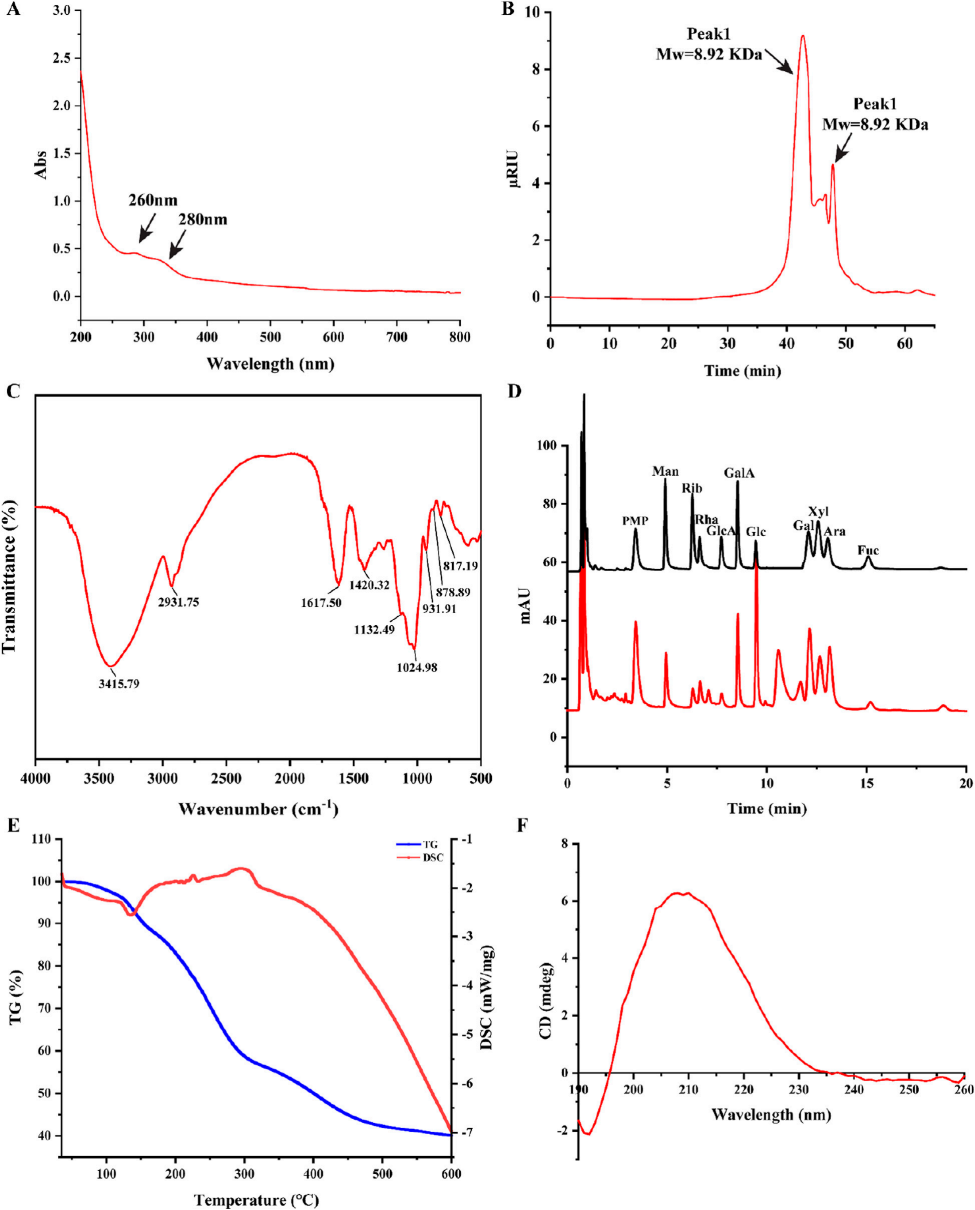
Structural characterization of MP. **(A)** UV spectra of MP; **(B)** Molecular weight of MP; **(C)** FT-IR spectra of MP; **(D)** The UPLC chromatograms of standard monosaccharides and MP; **(E)** Thermal stability, TG-DTG curve and DSC curve of MP; **(F)** CD spectra of MP.

The MP UV spectrum curve was shown in Figure 1A. The absorption peaks at wavelengths of 260 nm and 280 nm indicate a weak signal, suggesting a small amount of protein and nucleic acid in MP.

### 3.3 Determination of molecular weight distribution

The polysaccharides Mw (molecular weight) was strongly related to the biological activity of polysaccharides (Lee et al., 2024). The average Mw and homogeneity of MP was determined by HPGPC. Two peaks were observed in the chromatogram for MP (Figure 1B), with the average molecular weight of 8.92 kDa and 697 Da, separately. In addition, Peak Area of two peaks were 81.74% and 18.26%, respectively. This suggests that MP could be homogeneous after dialyzed (cut off Mw 3,000 Da) with distilled water.

### 3.4 FT-IR spectroscopy

The FT-IR spectroscopy is commonly employed to identify characteristic functional groups of polysaccharides. As shown in Figure 1C, the broad band at around 3,415 cm^-1^ can be assigned to the -OH stretching vibration, whereas the band at 2,931 cm^-1^ was related to the C-H stretching in the sugar ring. Futhermore, the characteristic absorption bands at approximately 817 and 878 cm^-1^ were attributed to the existence of the *α*-pyranose and *β*-pyranose configuration.

### 3.5 Monosaccharide composition

The UPLC chromatograms of standard monosaccharides and MP were indicated in Figure 1D. The analysis revealed that MP primarily consisted of ten monosaccharides: galactose, arabinose, xylose, fucose, glucuronic acid, galacturonic acid, rhamnose, glucose, ribose and mannose, the percentages was 17.40%, 13.92%, 16.85%, 1.92%, 15.67%, 1.63%, 4.00%, 18.36%, 2.91%, 7.72%, respectively.

### 3.6 Thermal characteristics

The thermal stability of MP was investigated by differential scanning calorimetry (DSC) and thermos gravimetry (TG) methods (Figure 1E). The TG curve exhibited three notable mass losses occurring between 35°C and 600°C. The first mass loss occurred between the temperature range of 35°C–140°C, with a rate of approximately 10%. Besides, an endothermic peak at 135°C on the DSC curve. This may be due to the evaporation of moisture from the sample. The second significant mass loss, occurring between 140°C and 450°C, corresponded to a substantial decrease in mass, likely resulting from the depolymerization of the polysaccharide structure. The region between 160°C and 450°C showed a mass loss rate of 45%. These results ultimately demonstrated that MP had good thermal stability.

### 3.7 Circular dichroism spectroscopy

The Circular dichroism spectrum curve of MP spanning from 190 nm to 260 nm was shown in Figure 1F, with a peak maximum observed at 210 nm.

### 3.8 Effects of MP treatments on colitis mice

**FIGURE 2:**
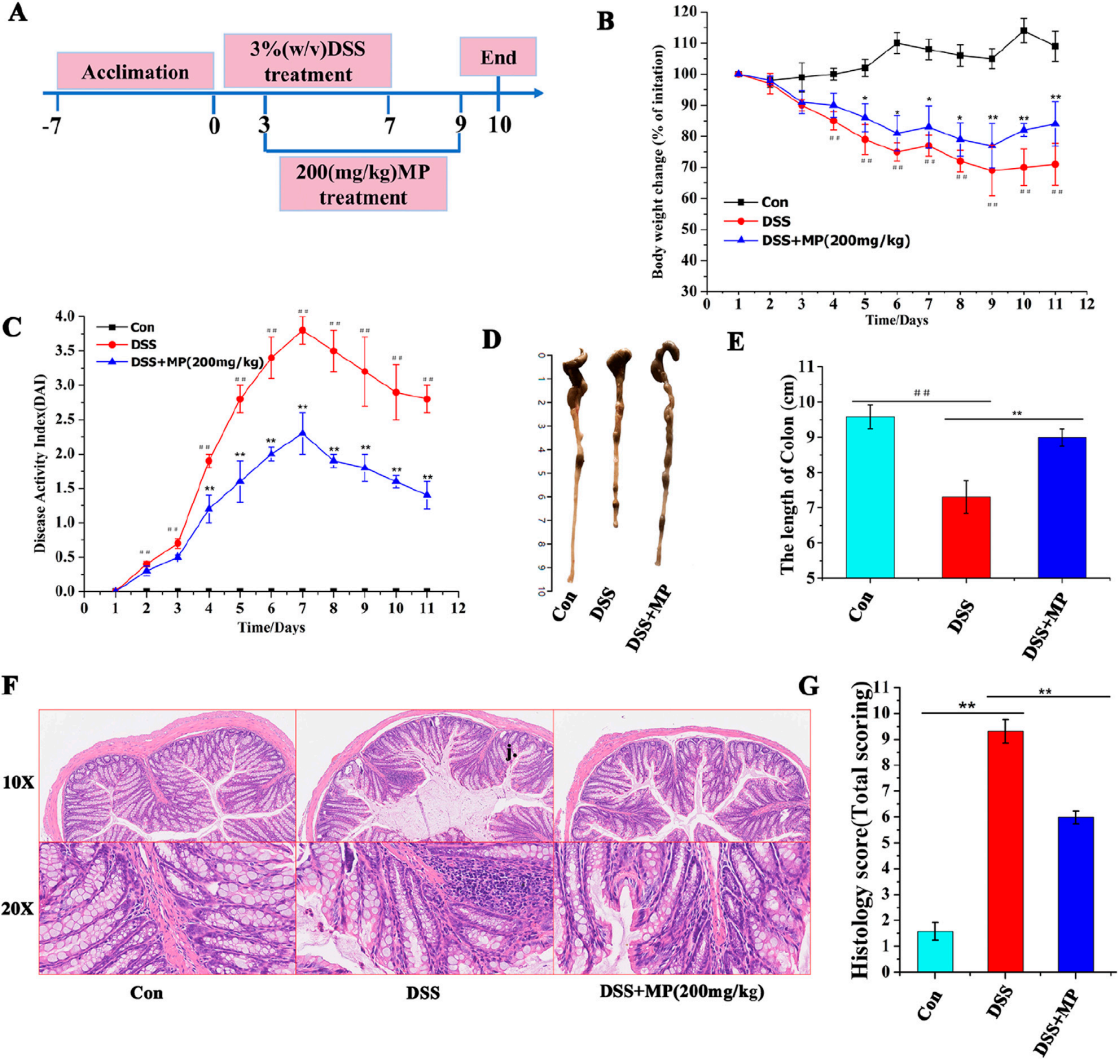
Oral MP alleviated DSS-induced experimental colitis. **(A)** The animal experimental protocol of this study. **(B)** Daily body weight changes throughout the entire duration of the study. (n = 8) **(C)** Kinetics of daily disease activity index (DAI) scores throughout the entire duration of the study. (n = 8) **(D)** Macroscopic pictures of colons and **(E)** the lengths of colon from each group. (n = 8). **(F)** H&E stained colon sections and **(G)** histological scores of colons (n = 5). Data were presented as means ± SEM. Statistical significance was determined using one-way ANOVA, followed by Tukey test. *P ≤ 0.05, **P ≤ 0.01.

To study the effects of MP treatments on the development of ulcerative colitis, a highly reproducible DSS-induced colitis model was employed (Li et al., 2022). Mice were given 3.5% DSS for 7 days, and C57BL/6 mice received oral MP between days 3 and 9 (Figure 2A). Administration of MP (200 mg/kg) observably mitigated the body weight loss induced by DSS when compared to the model group (Figure 2B). In this study, oral MP was administered to mice via oral gavage. Specifically, MP was dissolved in water and then administered to the mice through oral gavage with a volume of 0.2 mL. As depicted in Figure 2C, compared to the DSS group, oral MP intervention markedly alleviated DSS-induced colitis. This was evident from a substantial decrease in DAI (disease activity index), a composite score considering stool consistency, body weight loss, and rectal bleeding. Additionally, MP treatment markedly restrained the shortening of colitis mice colon length (Figures 1D,E). Moreover, the treatment considerably reduced mucosal damage and inflammatory cell infiltration (Figure 2F), thereby improving the overall histological score of the colon (Figure 2G). In summary, these findings indicate that MP exhibits potent anti-inflammatory effects.

### 3.9 Regulation of the PI3K/AKT signaling pathway

**FIGURE 3:**
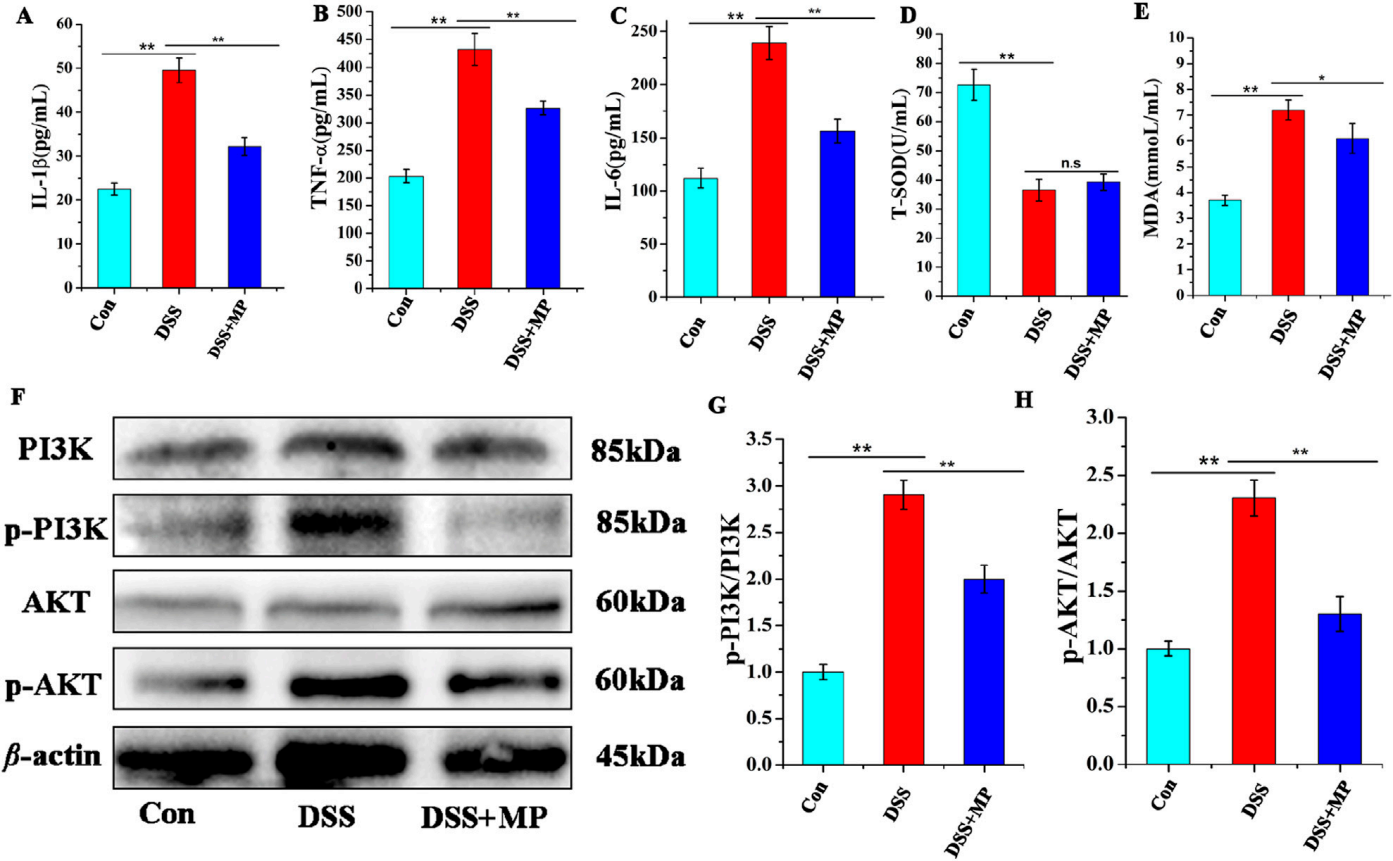
Concentrations of three representative pro-inflammatory cytokines, IL-1β **(A)**, TNF-α **(B)**, and IL-6 **(C)** in the serum. Concentrations of (T-SOD) **(D)**, and MDA **(E)** in the serum (n = 8). Effects of MP on the colon protein expression levels in mice **(F)**. The protein expression of p-AKT,AKT,p-PI3K and PI3K. Quantitative analysis of p-PI3K **(G)**, and p-AKT **(H)** (n = 3). Data were presented as means ± SEM. Statistical significance was determined using one-way ANOVA, followed by Tukey test. *P ≤ 0.05, **P ≤ 0.01.

To further evaluate the influence of MP on the response of intestinal inflammation, the plasma concentrations of pro-inflammatory cytokines were measured. Upon oral MP administration, the plasma levels of IL-1β (Figure 3A), TNF-α (Figure 3B), and IL-6 (Figure 3C) were notably reduced in DSS-treated mice. However, MP treatment did not significantly decrease the levels of T-SOD (Figure 3D) and MDA in serum (Figure 3E). These findings collectively indicate the apparent anti-inflammatory effects of MP. Furthermore, Western blot analysis was conducted to examine the expression levels of associated proteins involved in the PI3K/AKT signaling pathway (He et al., 2021; Xue et al., 2021; Chen et al., 2022). Notably, In the model group, the expression levels of p-PI3K and p-AKT (Figure 3F) were markedly elevated contrasts with that those in the control and MP groups. These findings indicate that MP can suppress the activation of the PI3K signaling pathway (Figures 3G,H).

### 3.10 Modulation of the gut microbiome and SCFAs by MP

**FIGURE 4:**
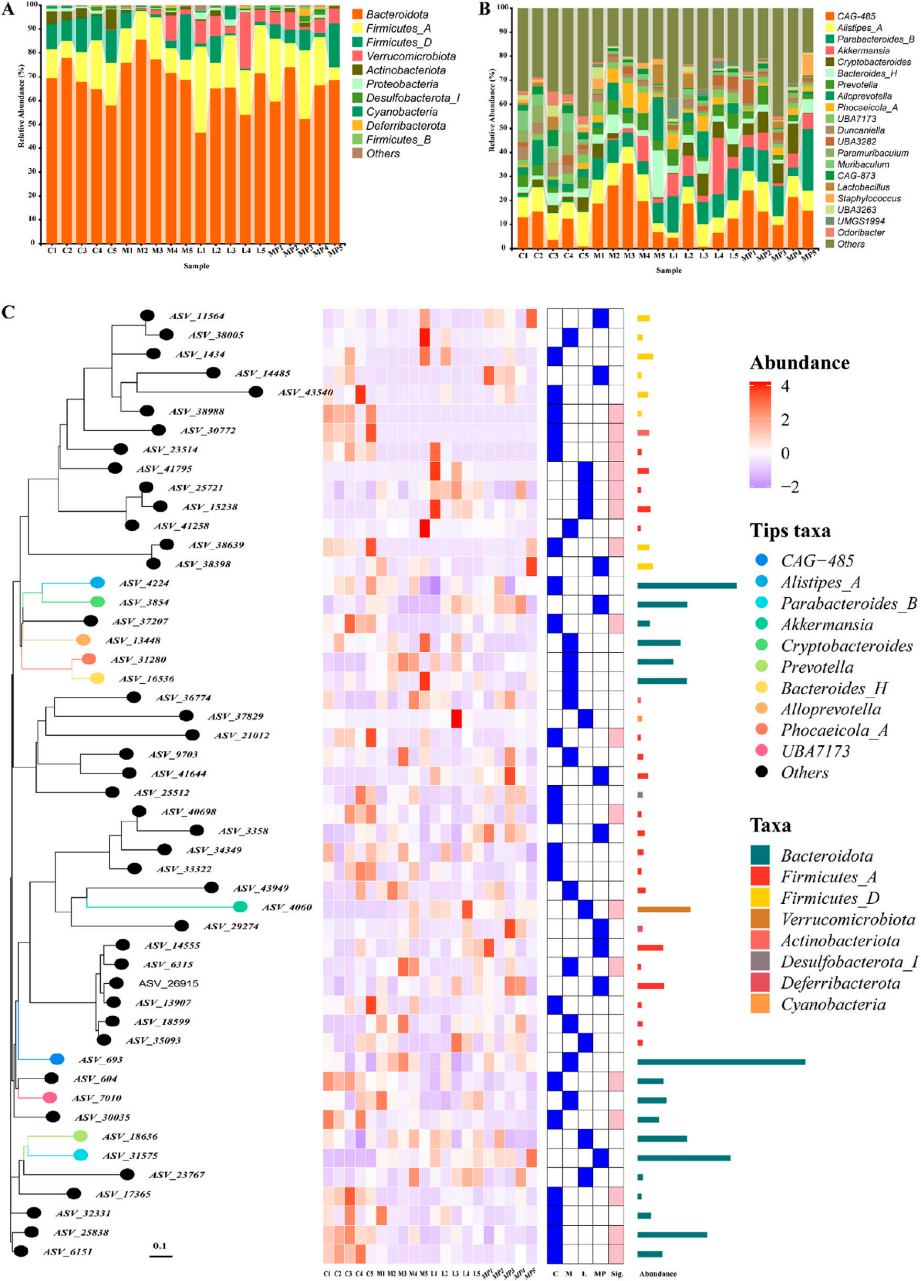
Modulation of the gut microbiome composition by MP: **(A)** gut microbiome composition at the phylum level, **(B)** gut microbiome composition at the genus level, **(C)** PhyloTree of gut microbiome composition.

The gut microbiome includes a complex colonisation of micro-organisms in the gut that plays an important role for the host physical condition (Weersma et al., 2020; De Vos et al., 2022; Gacesa et al., 2022). The gut microbiome dysbiosis has been closely associated with the emergence and advancement of IBD (Pittayanon et al., 2020; Shan et al., 2022; Li et al., 2023). As shown in Figure 4A, at the phylum level, Bacteroidota and Firmicutes-A were the two predominant microbiota phyla, and it suggested that DSS administration might lead to an upregulation in Bacteroidota and Firmicutes-A. Following MP administration, the richness of Bacteroidota and Firmicutes-A returned to normal levels comparable to those observed in the control and positive control groups. Bacteroidota is the hugest Gram-negative phylum of gut microbiome. Typically, it is beneficial to the host physical condition when confined within the gut. It was able to reduce the Firmicutes/Bacteroidetes (F/B) ratio (Zhao et al., 2020; Wolter et al., 2021; Fishbein et al., 2023), leading to an alleviation of colitis. After establishing the regulatory function of MP in the gut microbiome at the phylum level, the bacteria that might explain its effects on chronic colonic inflammation in mice at the genus level were further investigated. As shown in the relative richness graph (Figure 4B), CAG-485 and Alistipes-A were immensely enhanced in the DSS administration group in contrast to the control and positive control group, and the MP treatment obviously diminished its richness as the positive control. According to the PhyloTree (Figure 4C), the position of each ASV/OTU in the evolutionary tree and the evolutionary distance between each other were shown, and their composition, abundance, taxonomy were clearly reflected through heat maps and bar charts.

**FIGURE 5:**
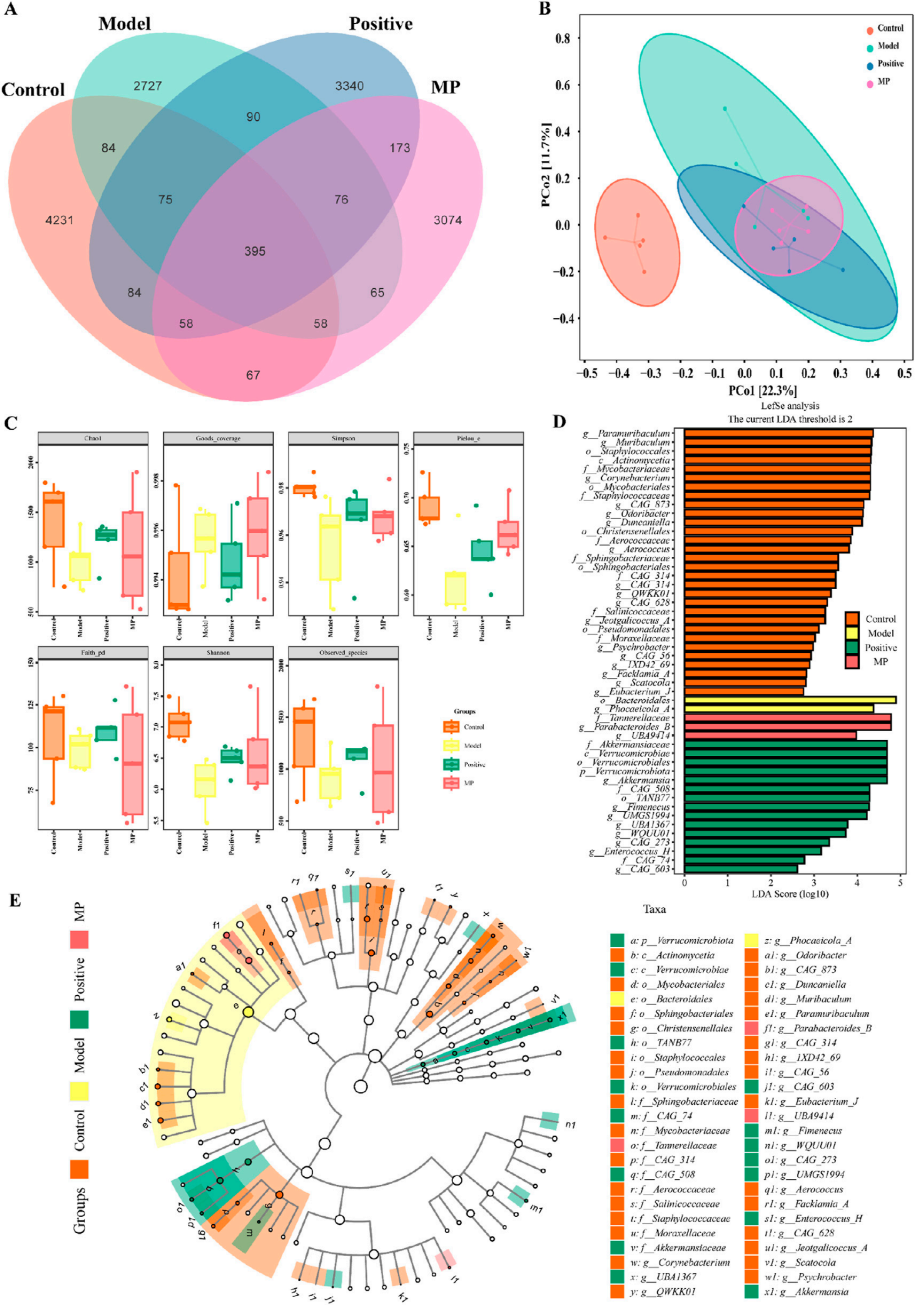
Modulation of the gut microbiome diversity by MP: **(A)** Venn diagram of gut microbiome, **(B)** β-diversity of gut microbiome component in varying groups, **(C)** α-diversity gut microbiome component in varying groups, **(D)** Linear discriminant analysis effect size comparative analysis, **(E)** linear discriminant analysis.

According to Figure 5A, the Venn diagram revealed that 4,231, 2727,3340, and 3,074 sole OTUs were identified in the control group, model group, positive control group and MP group, respectively. Based on the level of OTU, the PCoA (principal coordinate analysis) was employed to evaluate the β-diversity of gut microbiome component in varying groups (Figure 5B).

For the assess of impact on species diversity, alpha diversity investigation was conducted. Figure 5C present that goods-coverage index, Shannon index, chao one index, Simpson index, pielou-e index, faith-pd index, and obtained species index revealed the multiplicity of the gut microbiome configuration were decreased in model group contrasted with the control positive control group. However, MP group and positive control reversed this result. LDfSe (Linear discriminant analysis effect size) (O’Donnell et al., 2023) comparative analysis and LDA (linear discriminant analysis) (Cryan et al., 2020) were utilized to assess the impact of MP on the gut microbiome composition across the four groups (Figures 5D,E). The results showed that Bacteroidales and Phocaeicola-A were predominant bacteria in DSS-induced mice (model group), whereas Tannerellaceae, Parabacteroides-B and UBA9414 were recognized as predominant bacteria in mice with MP treatment (MP group).

**FIGURE 6:**
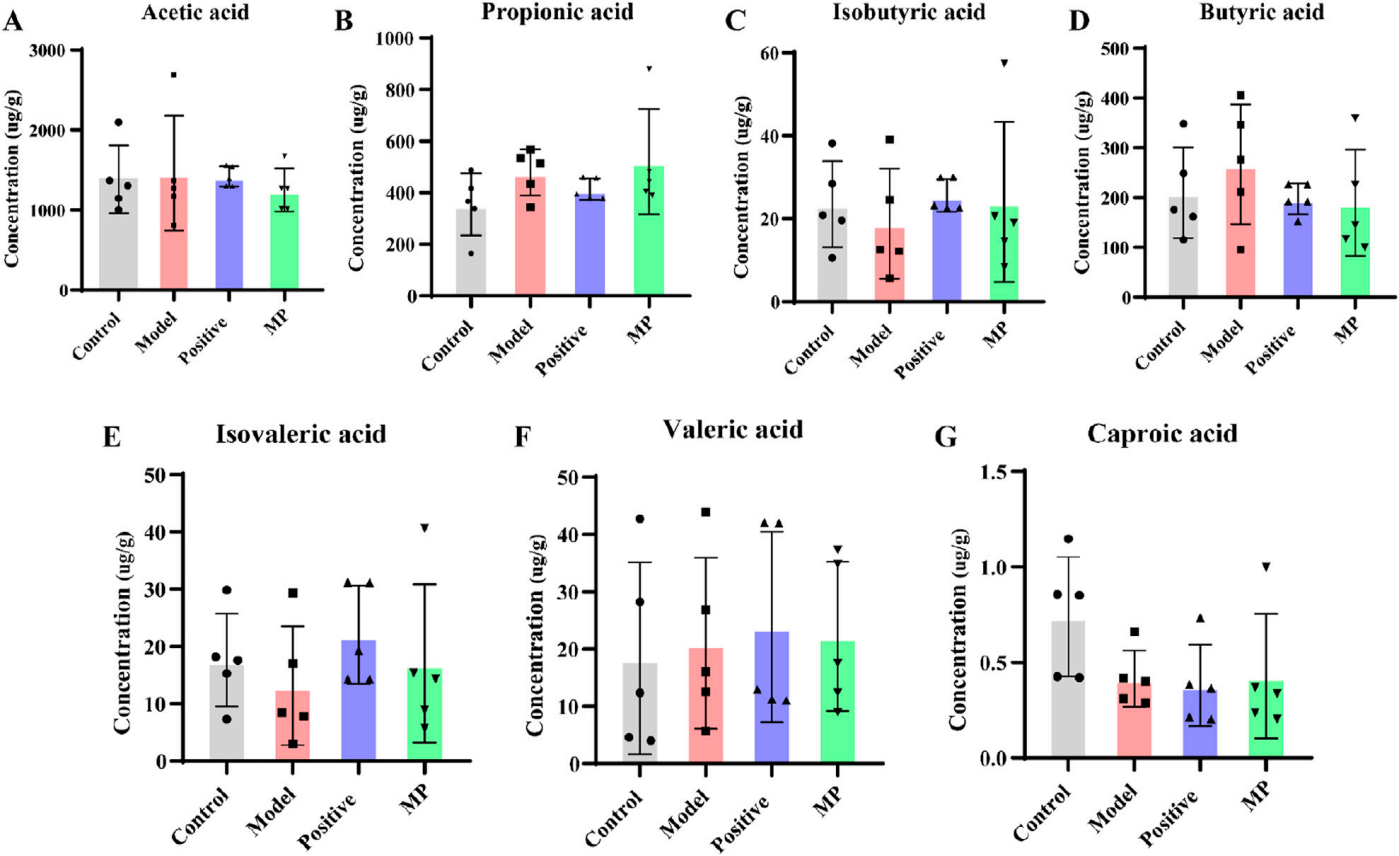
Regulation of SCFAs contents by MP: **(A)** concentration of acetic acid, **(B)** concentration of propionic acid, **(C)** concentration of isobutyric acid, **(D)** concentration of butyric acid, **(E)** concentration of isovaleric acid, **(F)** concentration of valeric acid, and **(G)** concentration of caproic acid.

SCFAs are metabolites derived from gut microbiota that play crucial roles in maintaining normal intestinal morphology and function (Frampton et al., 2020; Martin-Gallausiaux et al., 2021; van der Hee and Wells, 2021). The concentrations of SCFAs across each group were displayed in Figures 6A–G. In contrast to the control group, the concentration of butyrate, propionic acid, valeric acid and butyric acid were obviously elevated, while the concentration of isovaleric, isobutyric acid, and caproic acid notably decreased in the model group. MP treatment normalized these seven SCFAs to within normal levels.

## 4 Discussion

The current study elucidates the therapeutic potential of MP in alleviating DSS-induced colitis in mice. Our findings highlight the efficacy of MP in mitigating colitis symptoms, suggesting a novel natural remedy for the treatment of IBD. MP not only exhibited anti-inflammatory effects by suppressing the PI3K/AKT signaling pathway but also modulated the gut microbiome diversity and SCFA concentrations, contributing to an improved intestinal microenvironment.

Our results demonstrate that MP significantly alleviated DSS-induced colitis in mice, as evidenced by reduced weight loss, DAI scores, colon shortening, and tissue damage. These findings are comparable to those observed with other therapeutics used for colitis alleviation, such as aminosalicylates, anti-TNF-α agents, and JAK inhibitors (Pittayanon et al., 2020; Adolph et al., 2022). However, MP has the advantage of being a natural product with minimal side effects, making it a promising alternative to synthetic drugs. The predicted mechanism of action of MP involves the inhibition of the PI3K/AKT signaling pathway, which is a critical pathway involved in the regulation of inflammatory responses. This pathway is dysregulated in IBD, leading to excessive inflammation and tissue damage (Chen et al., 2022). By suppressing this pathway, MP may reduce the production of pro-inflammatory cytokines, such as IL-1β, TNF-α, and IL-6, thereby alleviating colitis symptoms. In comparison to FDA-approved medications for IBD, MP may offer a more natural and holistic approach to treatment. While synthetic drugs are highly effective, they often come with significant side effects. MP, on the other hand, has the potential to provide similar therapeutic benefits with minimal side effects. Furthermore, MP may be combined with other medications to enhance their efficacy and reduce side effects.

The modulation of gut microbiome diversity and SCFA concentrations by MP is another important aspect of its anti-inflammatory effects. The gut microbiome plays a crucial role in maintaining intestinal homeostasis, and its dysbiosis has been implicated in the pathogenesis of IBD (Shan et al., 2022). MP promoted the growth of beneficial bacteria, such as Tannerellaceae and Parabacteroides-B, while inhibiting the growth of harmful bacteria, such as CAG-485 and Alistipes-A. These changes in gut microbiome composition may contribute to the improved intestinal microenvironment observed in MP-treated mice. In addition, MP normalized the concentrations of SCFAs, such as butyrate, propionic acid, and valeric acid, which are important for maintaining intestinal health. SCFAs are metabolites derived from gut microbiota that play crucial roles in regulating inflammatory responses and maintaining intestinal barrier integrity (van der Hee and Wells, 2021). By modulating the gut microbiome and SCFA concentrations, MP may contribute to the alleviation of colitis symptoms through multiple mechanisms.

Our study highlights the novel therapeutic potential of MP in the treatment of IBD. The ability of MP to suppress the PI3K/AKT signaling pathway, modulate the gut microbiome diversity, and regulate SCFA concentrations makes it a promising candidate for the development of novel therapeutic strategies. Future studies should aim to further elucidate the mechanisms underlying the anti-inflammatory effects of MP and investigate its efficacy in clinical trials. In conclusion, our findings emphasize the importance of MP in the treatment of DSS-induced colitis and open up new avenues for targeted therapeutic approaches in the therapy of IBD. The modulation of gut microbiome diversity and SCFA concentrations by MP may represent a novel mechanism of action that can be exploited for the development of new therapeutic agents.

## 5 Conclusion

This work extensively studied the alleviation of *M*. *polysaccharides* for DSS-induced mice colitis. Firstly, *M*. *polysaccharides* were primarily characterized by chemical composition, homogeneity molecular weight, monosaccharide composition and thermal analysis. Then, its colitis alleviation activity was confirmed by *in vivo* experiments. The action mechanism was further primarily reveled by cell model and gut microbiota diversity. And it was found that *M*. *polysaccharides* could alleviate DSS-induced mice colitis through PI3K/AKT signaling pathway. In addition, it alleviated the advancement of colitis by enhancing the regulation of gut diversity and concentration of short-chain fatty acids. Our results emphasize the greater efficacy of *M*. *polysaccharides* in relieving DSS-induced colitis and open up new way for targeted therapeutic approaches in the therapy of IBD.

## Data Availability

The datasets presented in this study can be found in online repositories. The names of the repository/repositories and accession number(s) can be found below: PRJNA1134356 (SRA).
